# Sensitivity to Neutralizing Antibodies and Resistance to Type I Interferons in SARS-CoV-2 R.1 Lineage Variants, Canada 

**DOI:** 10.3201/eid2907.230198

**Published:** 2023-07

**Authors:** Rajesh Abraham Jacob, Ali Zhang, Hannah O. Ajoge, Michael R. D'Agostino, Kuganya Nirmalarajah, Altynay Shigayeva, Wael L. Demian, Sheridan J.C. Baker, Hooman Derakhshani, Laura Rossi, Jalees A. Nasir, Emily M. Panousis, Ahmed N. Draia, Christie Vermeiren, Jodi Gilchrist, Nicole Smieja, David Bulir, Marek Smieja, Michael G. Surette, Andrew G. McArthur, Allison J. McGeer, Samira Mubareka, Arinjay Banerjee, Matthew S. Miller, Karen Mossman

**Affiliations:** McMaster University, Hamilton, Ontario, Canada (R.A. Jacob, A. Zhang, H.O. Ajoge, M.R. D'Agostino, W.L. Demian, S.J.C. Baker, L. Rossi, J.A. Nasir, E.M. Panousis, A.N. Draia, D. Bulir, M. Smieja, M.G. Surette, A.G. McArthur, M.S. Miller, K. Mossman);; Sunnybrook Research Institute, Toronto, Ontario, Canada (K. Nirmalarajah, S. Mubareka);; University of Toronto, Toronto (A. Shigayeva, C. Vermeiren, A.J. McGeer, S. Mubareka, A. Banerjee);; University of Manitoba, Winnipeg, Manitoba, Canada (H. Derakhshani);; Research Institute of St. Joe’s Hamilton, Hamilton (J. Gilchrist, N. Smieja, D. Bulir);; Vaccine and Infectious Disease Organization, Saskatoon, Saskatchewan, Canada (A. Banerjee);; University of Saskatchewan, Saskatoon (A. Banerjee);; University of Waterloo, Waterloo, Ontario, Canada (A. Banerjee);; University of British Columbia, Vancouver, British Columbia, Canada (A. Banerjee)

**Keywords:** COVID-19, SARS-CoV-2, coronavirus disease, severe acute respiratory syndrome coronavirus 2, viruses, respiratory infections, zoonoses, variant under monitoring, VuM, variants of concern, VoC, N_501_Y spike, neutralizing antibodies, type I interferons, Canada

## Abstract

Isolating and characterizing emerging SARS-CoV-2 variants is key to understanding virus pathogenesis. In this study, we isolated samples of the SARS-CoV-2 R.1 lineage, categorized as a variant under monitoring by the World Health Organization, and evaluated their sensitivity to neutralizing antibodies and type I interferons. We used convalescent serum samples from persons in Canada infected either with ancestral virus (wave 1) or the B.1.1.7 (Alpha) variant of concern (wave 3) for testing neutralization sensitivity. The R.1 isolates were potently neutralized by both the wave 1 and wave 3 convalescent serum samples, unlike the B.1.351 (Beta) variant of concern. Of note, the R.1 variant was significantly more resistant to type I interferons (IFN-α/β) than was the ancestral isolate. Our study demonstrates that the R.1 variant retained sensitivity to neutralizing antibodies but evolved resistance to type I interferons. This critical driving force will influence the trajectory of the pandemic.

SARS-CoV-2 continues to evolve and generate new variants. Since the beginning of the COVID-19 pandemic, Canada has encountered 8 waves of infections. Although the first 2 waves were dominated by ancestral viruses, each subsequent wave had a surge in escape variants (*1*,*2*). Mutations in the SARS-CoV-2 genome and within the spike glycoprotein alter the transmission dynamics, severity of disease, and sensitivity to neutralizing antibodies for each new variant (*3*). Thus, continuously isolating and characterizing emerging SARS-CoV-2 variants is critical for developing updated vaccines and drug regimens.

We isolated SARS-CoV-2 R.1 lineage variants from an outbreak in persons facing housing insecurity in Hamilton, Ontario, Canada. In Canada, the circulation of R.1 lineage variants corresponded with the third wave of the pandemic and was preceded by previously circulating variants of concern (VoC), B.1.1.7 (Alpha) and B.1.351 (Beta). Globally, the R.1 lineage began to increase in frequency in December 2020, peaked in April 2021, became rare by June 2021, and was last reported in December 2021 (*4*). In April 2021, the World Health Organization positioned R.1 lineage variants under the variant under monitoring (VuM) category to prioritize monitoring the variants because of distinct mutations in their genome. Most infections with R.1 variants have been reported in Japan and the United States (*5*,*6*). In Canada, 66 R.1 lineage sequences were recorded, according to GISAID (https://www.gisaid.org), during December 2020–November 2021 (*4*). Of those, 63 originated from Ontario, 1 each originated from Quebec and British Columbia, and 1 originated from an unknown province or territory. However, data on the immune-evasive properties of this lineage variant are limited.

The type I interferon (IFN) response constitutes the first line of defense against many viruses (*5*,*6*), triggering activation of several IFN-stimulated genes (ISGs) and establishing an antiviral state (*5*). SARS-CoV-2 proteins are involved in IFN evasion either by directly suppressing production or by acting downstream of the host response machinery (*6*,*7*). A recent study compared multiple type I IFNs against diverse SARS-CoV-2 VoC, demonstrating increased IFN resistance (*8*). Furthermore, SARS-CoV-2–infected persons with genetic defects in IFN signaling are at higher risk for severe COVID-19 (*9*). Taken together, characterizing IFN-resistant SARS-CoV-2 variants is critical, given their potential to enhance transmission kinetics and result in viral evolution. Therefore, we evaluated the sensitivity of SARS-CoV-2 R.1 lineage isolates from patients in Canada to neutralizing antibodies and type I interferons.

## Methods

### Cells and Viruses

Vero E6 cells and Calu-3 cells were cultured in complete media (Appendix). We isolated and purified study isolate SB3 as described previously (*10*) and isolated the R.1 lineage variant from patient nasopharyngeal swab samples (Appendix). We obtained the B.1.351 (Beta) VoC isolate from BEI Resources (https://www.beiresources.org). 

### Human Donors

We obtained informed consent for the collection of convalescent serum samples from 39 patients with quantitative reverse transcription PCR (qRT-PCR)–confirmed SARS-CoV-2 infection (Appendix). This study was approved by the institutional review board for Sunnybrook Research Institute (approval no. 2218) and Sinai Health System (approval nos. 02-0118-U and 05-0016-C).

### SARS-CoV-2 Sequencing and Phylogenetic Tree 

We performed sequencing of SARS-CoV-2 genomes from RNA extracts and subsequent bioinformatics analysis following the steps detailed in Kotwa et al. (*11*) (Appendix). We constructed a maximum-likelihood phylogenetic tree using a dataset of study sequences (R.1 645, R.1 646, and SB3), Los Alamos National Laboratories full-length variant reference alignment from GISAID (*12*), and randomly sampled Alpha, Beta, and Gamma variant sequences (Appendix).

### Detection of SARS-CoV-2–Specific Binding Antibodies and Neutralization Assay 

We determined the IgG targeting the receptor-binding domain (RBD) and spike S1 region by using ELISA (BioLegend, https://www.biolegend.com) (Appendix). We performed neutralization assays by incubating serially diluted serum samples (heat inactivated) with SARS-CoV-2 (15,000, 1,500, or 150 PFU/well) at 37°C for 1 h before adding them to preplated Vero E6 cells. Five days after infection, we quantified luminescence with CellTiter-Glo 2.0 Reagent (Promega, https://www.promega.com) by using a BioTek Synergy H1 microplate reader (Appendix).

### Molecular Detection of SARS-CoV-2 N_501_Y Mutation 

Diagnostic nasopharyngeal or midturbinate swab specimens were collected from patients for SARS-CoV-2 testing and N_501_Y screening at Shared Hospital Laboratory (Toronto, Canada). We performed RNA extraction and qRT-PCR to detect SARS-CoV-2 as previously described (*13*) (Appendix).

### Interferon Treatment and Quantitative PCR 

Calu-3 cells were either mock-infected or SARS-CoV-2–infected (1 h exposure, 50,000 PFU/well), washed twice with sterile 1× phosphate-buffered serum, and treated with 1 ng/mL or 10 ng/mL of recombinant IFN-α (Sigma-Aldrich, https://www.sigmaaldrich.com) or IFNβ (PeproTech, https://www.peprotech.com). We quantified SARS-CoV-2 RNA and ISGs 72 h postinfection by using qRT-PCR (Appendix). 

### Viability Assay, Microscopy, and Statistical Analysis

We assessed cell viability of SARS-CoV-2–infected Calu-3 cells by using CellTiter-Glo 2.0 Reagent (Promega) (Appendix) and imaged SARS-CoV-2–infected Calu-3 cells for cytopathic elects using an EVOS M5000 microscope (ThermoFisher Scientific, https://www.thermofisher.com) with the 10× objective. We performed all statistical analyses by using GraphPad Prism (https://www.graphpad.com) (Appendix).

## Results

### R.1 Lineage Variant Virus Isolation and Lineage Determination

**Figure 1:**
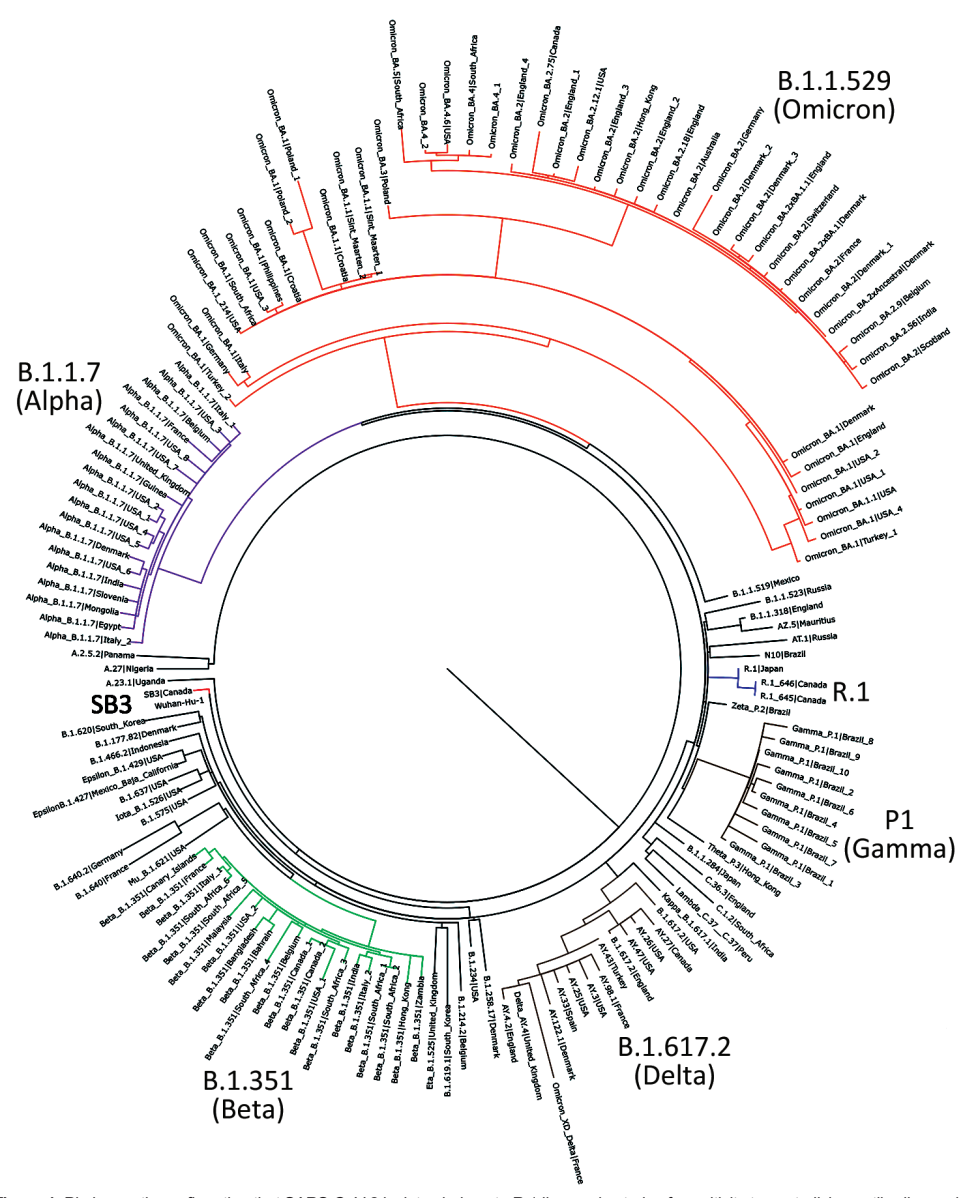
Phylogenetic confirmation that SARS-CoV-2 isolates belong to R.1 lineage in study of sensitivity to neutralizing antibodies and resistance to type I interferons in SARS-CoV-2 R.1 lineage variants, Canada. Tree constructed by using maximum-likelihood estimations by executing 1,000 rapid bootstrap inferences and a thorough search with the general time reversible model of nucleotide substitution. Blue indicates R1 isolates (R.1 645, R.1 646) and red SB3 isolate. Variants are highlighted in magenta (Alpha), green (Beta), brown (Delta), mocha (Gamma), and orange (Omicron). The tree was visualized using FigTree version 1.4.2 (http://tree.bio.ed.ac.uk/software/figtree).

We isolated 2 SARS-CoV-2 isolates belonging to the R.1 lineage (R.1 645, R.1 646) from 2 patients from Hamilton, Ontario, Canada, in March 2021, corresponding to the third wave of the COVID-19 pandemic in Canada. The 2 R.1 isolates were purified from nasopharyngeal samples from SARS-CoV-2–specific qRT-PCR–positive persons by using Vero clone E6 cells as described previously (*10*). We determined the viral whole-genome sequences and deposited them into GISAID (accession nos. EPI_ISL_16641180 and EPI_ISL_16641181). We performed phylogenetic analysis to confirm clustering with the R.1 lineage. The R.1 isolates clustered on their own distinctly from B.1.1.7 (Alpha), B.1.351 (Beta), B.1.617.2 (Delta), P.1 (Gamma), and B.1.1.529 (Omicron) variants (Figure 1). Sequencing of both R.1 isolates revealed multiple mutations in spike (W_152_L, S_255_F, E_484_K, D_614_G, G_769_V), nonstructural protein (NSP) 2 (P_129_L, A_247_V), NSP3 (S_1656_A), NSP12 (P_323_L), NSP13 (G_439_R, P_323_L), NSP14 (P_412_H), ORF3a (R_134_H), membrane (F_28_L), and the nucleocapsid (S_187_L, R_203_K, G_204_R, Q_418_H) regions critical to immune evasion (*14*).

### Wave 1 and Wave 3 Serum Samples

We next characterized the R.1 isolates to identify potential effects on the epidemiology of COVID-19. We assessed the susceptibility of R.1 isolates to neutralizing antibodies after SARS-CoV-2 infection in a cohort of patients who had recovered from laboratory-confirmed COVID-19 by collecting convalescent serum samples from 39 unvaccinated donors during the first (n = 26) and third (n = 13) waves of the pandemic in Canada. The cohort consisted of 20 men and 19 women with a median age of 58. Wave 1 consisted of 13 men and 13 women with a median age of 63. Wave 3 consisted of 7 men and 6 women with a median age of 53. Samples were collected a median of 42 days after the first qRT-PCR–positive test. Most patients were symptomatic and more than one third were hospitalized; a subset of hospitalized patients was admitted to the intensive care unit (Table 1). 

**Table 1:** Clinical summary of patients from study of sensitivity to neutralizing antibodies and resistance to type I interferons in SARS-CoV-2 R.1 lineage variants, Canada*

Characteristic	Value
Total no. samples	39
No. samples, wave 1	26
No. samples, wave 3	13
Median age, total (IQR)	58 (46–68)
Median age, wave 1 (IQR)	63 (55–71)
Median age, wave 3 (IQR)	53 (33–56)
Sex, total	
M	20
F	19
Sex, wave 1	
M	13
F	13
Sex, wave 3	
M	7
F	6
Median no. days to sampling from first COVID-19 positive test (IQR)	42 (25–71)
Hospital admission	14 (36)
Median days from symptom onset to admission (IQR)	6 (2–10)
Symptoms	
Asymptomatic	1 (2)
Fever	17 (43)
Cough	19 (54)
Shortness of breath	8 (20)
Comorbidities	
Diabetes	9 (23)
Cardiac illnesses	4 (10)
Vascular illnesses	13 (33)
Pulmonary illnesses	3 (8)
Renal illnesses	2 (5)
Neuromuscular illnesses	0 (0)
Liver illnesses	0 (0)
Gastrointestinal illnesses	0 (0)
Cancer conditions	3 (7)
Rheumatologic illnesses	1 (2)
Mental health diagnosis	3 (7)
Immunocompromised	0 (0)
ICU admission	4 (10)
Intubation	2 (5)

*Values are no. (%) except as indicated. ICU, intensive care unit; IQR, interquartile range.

Wave 1 (March–June 2020) was dominated by infection with the SARS-CoV-2 ancestral strain, whereas wave 3 (March–May 2021) was dominated by infection with the B.1.1.7 (Alpha) VoC (*2*,*15*). A key amino acid change in the RBD of the B.1.1.7 (Alpha) VoC was the N_501_Y substitution (*16*). We initially used qRT-PCR screening to confirm the presence of the N_501_Y mutation in the 13 clinical samples from wave 3 patients. The N_501_Y substitution enhances the affinity of RBD to the angiotensin-converting enzyme 2 receptor (*17*,*18*) and is present in the B.1.1.7 (Alpha), B.1.351 (Beta), P.1 (Gamma), and B.1.1.529 (Omicron) lineages but not in the R.1 lineage variants (*19*–*21*). Whole-genome sequencing confirmed that the wave 3 variant with the N_501_Y substitution belonged to the B.1.1.7 (Alpha) VoC. For this investigation, we elicited antibodies from wave 1 serum samples (n = 26) by infection with viruses harboring the ancestral SARS-CoV-2 spike, whereas we elicited antibodies from wave 3 serum samples (n = 13) by viruses harboring the B.1.1.7 (Alpha) VoC spike.

### Determination of SARS-CoV-2 Spike- and RBD-Binding Antibodies

**Figure 2:**
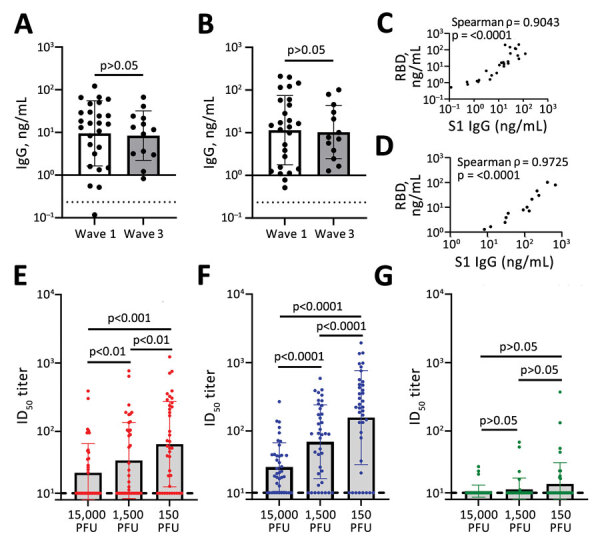
Antibody detection in study of sensitivity to neutralizing antibodies and resistance to type I interferons in SARS-CoV-2 R.1 lineage variants, Canada. A, B) S1 (A) and RBD (b) binding IgG determined by using a sandwich ELISA format. Dashed line indicates the limit of detection. C, D) Correlation between S1 and RBD binding IgG for wave 1 (C) and wave 3 (D). E–G) ID_50_ titers for SB3 (E), R.1 645 (F), and B.1.351 (Beta) VoC (E). Error bars in panels A, B and E–G indicate SD. Statistical significance was calculated by using an unpaired t test for panels A and B and by using 1-way analysis of variance with Tukey multiple comparisons test for panels E–G. ID_50_, 50% inhibitory dilution; PFU, plaque-forming units; RBD, receptor-binding domain; S1, spike.

Initially, we determined the binding ability of waves 1 and 3 serum samples to SARS-CoV-2 ancestral antigens using ELISA. We detected binding antibodies targeting the SARS-CoV-2 spike (S1 subunit) and RBD. Plates coated with either the ancestral S1 or the ancestral RBD were used to detect binding IgG. Antibodies from waves 1 and 3 serum samples bound the ancestral S1 and ancestral RBD (Figure 2, panels A, B). IgG levels from wave 1 serum samples were comparable to wave 3 serum samples for both the S1 (geometric mean 9.51 vs. 8.42 ng/mL) and the RBD (geometric mean 11.47 vs. 10.30 ng/mL) (Figure 2, panels A and B). This observation indicates that binding antibodies generated by the B.1.1.7 (Alpha) VoC in wave 3 were cross-reactive with the ancestral S1 and ancestral RBD. 

Next, we investigated the correlation between S1-targeting IgG and the RBD-targeting IgG. The S1 and the RBD targeting IgG correlated well; for both wave 1 (Spearman ρ = 0.9043; p<0.0001) and wave 3 serum samples (Spearman ρ = 0.9725; p<0.0001) (Figure 2, panels C. D). This correlation indicates that for both wave 1 and wave 3 serum samples, the ancestral S1 and RBD antigens were equally available for binding.

### Neutralization of SB3, R.1 645, and B.1.351 (Beta) VoC

Next, we assessed the antibody function by using a neutralization assay for the entire cohort. We compared the neutralization sensitivity of the R.1 isolates with SB3 and B.1.351 (Beta) VoC. Live SARS-CoV-2 isolates were used for the neutralization assay. SB3 is an ancestral isolate purified from a SARS-CoV-2–infected patient in early 2020 from Toronto, Canada (*10*). B.1.351 is a highly neutralization-resistant isolate that was first detected in late 2020 from Eastern Cape, South Africa (*22*). B.1.351 has extensive mutations in the spike region (L_18_F, D_80_A, D_215_G, Δ_242–244_, K_417_N, E_484_K, N_501_Y, D_614_G, and A_701_V), conferring resistance to antibodies from both convalescent and vaccinated persons (*22*–*26*). 

To establish a robust readout for neutralization, we tested 3 different plaque-forming unit (PFU) levels (15,000, 1,500, and 150) per well for all 3 isolates (SB3, R.1 645, and B.1.351 [Beta] VoC). We generated neutralization profiles for SB3 (Appendix Figure 1, panels A–C), R.1 645 (Appendix Figure 1, panels D–F), and B.1.351 (Appendix Figure 1, panels G–I) and derived 50% inhibitory dilution (ID_50_) values. For SB3, we observed a significant difference in ID_50_ between the 3 different PFUs tested; 150 PFU/well was the most neutralization sensitive and 15,000 PFU/well the most resistant (Figure 2, panel E). The geometric means of ID_50_ titers were 24.9 for 15,000 PFU/well, 37.7 for 1,500 PFU/well, and 65.2 for the 150 PFU/well (Figure 2, panel E). As for SB3, the geometric mean of R.1 645 ID_50_ titers increased from 29.7 for 15,000 PFU/well to 69.6 for 1,500 PFU/well and 156.9 for 150 PFU/well (Figure 2, panel F). However, for B.1.351, we noticed no significant difference among the 3 PFUs tested. The geometric mean of ID_50_ titers remained very low: 13.2 for 15,000 PFU/well, 14.0 for 1,500 PFU/well, and 16.9 for 150 PFU/well (Figure 2, panel G), corroborating previous data that B.1.351 is highly resistant to neutralization (*23*–*25*). This neutralization profile shows that the serum samples have diverse neutralizing abilities; titers significantly increased as the number of viral particles decreased for SB3 and R.1 645 but not for the highly resistant B.1.351.

### Sensitivity of R.1 645 and R.1 646 to Neutralizing Antibodies

Next, we determined whether the 2 R.1 isolates (R.1 645, R.1 646) had similar sensitivity to neutralizing antibodies by screening a subset of the serum samples (n = 19 from wave 1 and 3) on R.1 646 (Appendix Figure 2, panels A–C). The Spearman rank correlation coefficient (ρ) values remained high for 15,000 (Spearman ρ = 0.8333; p<0.0001), 1,500 (Spearman ρ = 0.9262; p<0.0001), and 150 (Spearman ρ = 0.8677; p<0.0001) PFU/well (Appendix Figure 2, panels D–F), indicating that the R.1 isolates were similarly neutralized by the serum samples.

### Binding IgG as a Prediction of SARS-CoV-2 Neutralization

We next assessed whether binding antibodies are predictive of SARS-CoV-2 neutralization. We used a linear regression model to predict whether the RBD and spike (S1) targeting IgG could neutralize SB3, R.1 645, and B.1.351 (Beta) VoC, using only the 150 PFU/well condition because it had the highest neutralization titers. RBD-binding IgG were a weak predictor of SB3 (R^2^ = 0.3819; p<0.0001) and R.1 645 (R^2^ = 0.2225; p = 0.0024) neutralization, but we noted no significance for B.1.351 (Beta) VoC (R^2^ = 0.05892; p>0.05) (Appendix Figure 3, panel A). In contrast to RBD, the neutralization prediction was moderately improved for S1-binding IgG for SB3 (R^2^ = 0.5148; p<0.0001) and R.1 645 (R^2^ = 0.6025; p<0.0001) but weak for B.1.351 (R^2^ = 0.1308; p<0.0001) (Appendix Figure 3, panel B). Those data suggest that S1-targeting antibodies outside the RBD are markedly involved in neutralization.

### Neutralization of R.1 Isolate by Wave 1 Serum Samples

**Figure 3:**
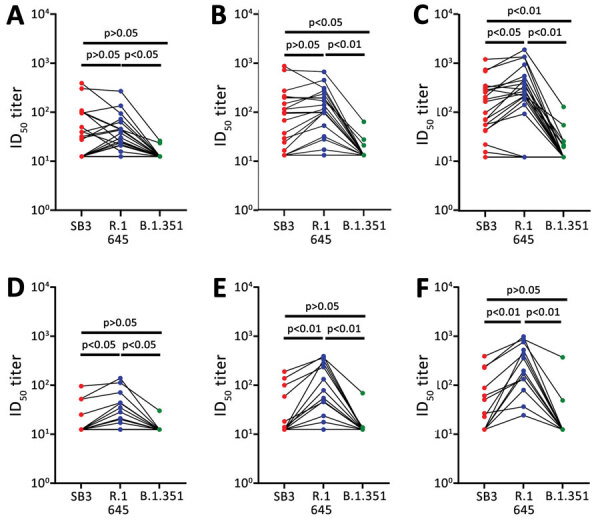
Sensitivity of SARS-CoV-2 lineage variants to neutralizing antibodies, Canada. A–C) Sensitivity of SB3, R.1 645, and B.1.351 (Beta) variants to neutralizing antibodies from patients infected with the ancestral virus (wave 1 samples). D–F) Sensitivity of SB3, R.1 645, and B.1.351 (Beta) VoC to neutralizing antibodies from patients infected with the B.1.1.7 (Alpha) VoC (wave 3 samples). For each isolate, we tested 3 different PFU per well: 15,000 (A, D), 1,500 (B, E), and 150 (C, F). Statistical significance was calculated using 1-way analysis of variance with Tukey multiple comparisons test. ID_50_, 50% inhibitory dilution; PFU, plaque-forming units.

Next, we determined whether the R.1 isolate (R.1645) that emerged during wave 3 was sensitive to neutralizing antibodies elicited by the ancestral virus from wave 1. To test this possibility, we compared the neutralization susceptibility of R.1 645 with SB3 and B.1.351 (Beta) VoC. We observed no significant difference in the ID_50_ titers between SB3 and R.1 645 at higher PFUs (15,000 and 1,500) (Figure 3, panels A, B). However, R.1 645 was significantly more sensitive than SB3 at 150 PFU/well; we noted a 1.6-fold increase in the geometric mean of the ID_50_ titers (Figure 3, panel C). Furthermore, R.1 645 was significantly more sensitive than B.1.351 at all 3 PFUs tested (Figure 3, panels A–C). A 2.2-fold, 4.5-fold, and 8.2-fold increase in the geometric mean of ID_50_ titers was noted for R.1 645 in comparison to B.1.351 (Figure 3, panels A–C). SB3 also remained significantly more sensitive than B.1.351 at lower PFUs (3.3-fold increase in geometric mean of ID_50_ titers at 1,500 PFU/well and 5-fold increase at 150 PFU/well) (Figure 3, panels B, C). This observation indicates that the R.1 isolate, despite having spike mutations, remains sensitive to antibodies from wave 1 serum samples (Appendix Table 2).

### Neutralization of R.1 Isolate by Wave 3 Serum Samples

Subsequently, we analyzed whether the antibodies elicited by the B.1.1.7 (Alpha) VoC during wave 3 of the pandemic can neutralize the R.1 isolate. A significant increase in the ID_50_ titers was notable between SB3 and R.1 645 at all the PFUs tested (Figure 3, panels D–F). We noted 1.6-fold, 3.5-fold, and 5.1-fold increases in the geometric mean of the ID_50_ titers for R.1 645 in comparison to SB3. This observation indicates that the antibodies triggered by the B.1.1.7 (Alpha) VoC could neutralize R.1 645 better than SB3. Neutralization titers of R.1 645 also remained high compared with B.1.351 (Beta) VoC, indicating that B.1.351 is significantly resistant to wave 3 serum samples (Figure 3, panels D–F). We noted 2.3-fold, 6.1-fold, and 11.9-fold increases in the geometric mean of ID_50_ titers for R.1 645 in comparison to B.1.351 (Figure 3, panels D–F). However, unlike wave 1 serum samples, we noted no significant difference in the ID_50_ titers between SB3 and B.1.351 for wave 3 serum samples (Figure 3, panels D–F). Those data indicate that the antibody repertoire evolved over time resulting in a substantial loss of neutralization breadth to the ancestral isolate. Of note, the antibody repertoire that evolved in response to the B.1.1.7 (Alpha) VoC during wave 3 could still neutralize the R.1 645 isolate (Table 2).

**Table 2:** Summary of neutralization of SARS-CoV-2 isolates from study of sensitivity to neutralizing antibodies and resistance to type I interferons in SARS-CoV-2 R.1 lineage variants, Canada

SARS-CoV-2 isolates	Sensitive isolate	Adjusted p value
Wave 1: 15,000 PFU		
SB3 vs. R.1 645	NA	NS
SB3 vs. B.1.351	NA	NS
R.1 645 vs. B.1.351	R.1645	0.0186
Wave 1: 1,500 PFU		
SB3 vs. R.1 645	NA	NS
SB3 vs. B.1.351	SB3	0.0243
R.1 645 vs. B.1.351	R.1 645	0.0011
Wave 1: 150 PFU		
SB3 vs. R.1 645	R.1 645	0.0141
SB3 vs. B.1.351	SB3	0.0066
R.1 645 vs. B.1.351	R.1 645	0.0022
Wave 3: 15,000 PFU		
SB3 vs. R.1 645	R.1 645	0.0391
SB3 vs. B.1.351	NA	NS
R.1 645 vs. B.1.351	R.1 645	0.0385
Wave 3: 1,500 PFU		
SB3 vs. R.1 645	R.1 645	0.0079
SB3 vs. B.1.351	NA	NS
R.1 645 vs. B.1.351	R.1 645	0.0093
Wave 3: 150 PFU		
SB3 vs. R.1 645	R.1 645	0.0038
SB3 vs. B.1.351	NA	NS
R.1 645 vs. B.1.351	R.1 645	0.0053

*NA, not applicable; NS, not significant; PFU, plaque-forming units.

### Sensitivity of R.1 Isolates to Type I Interferons

**Figure 4:**
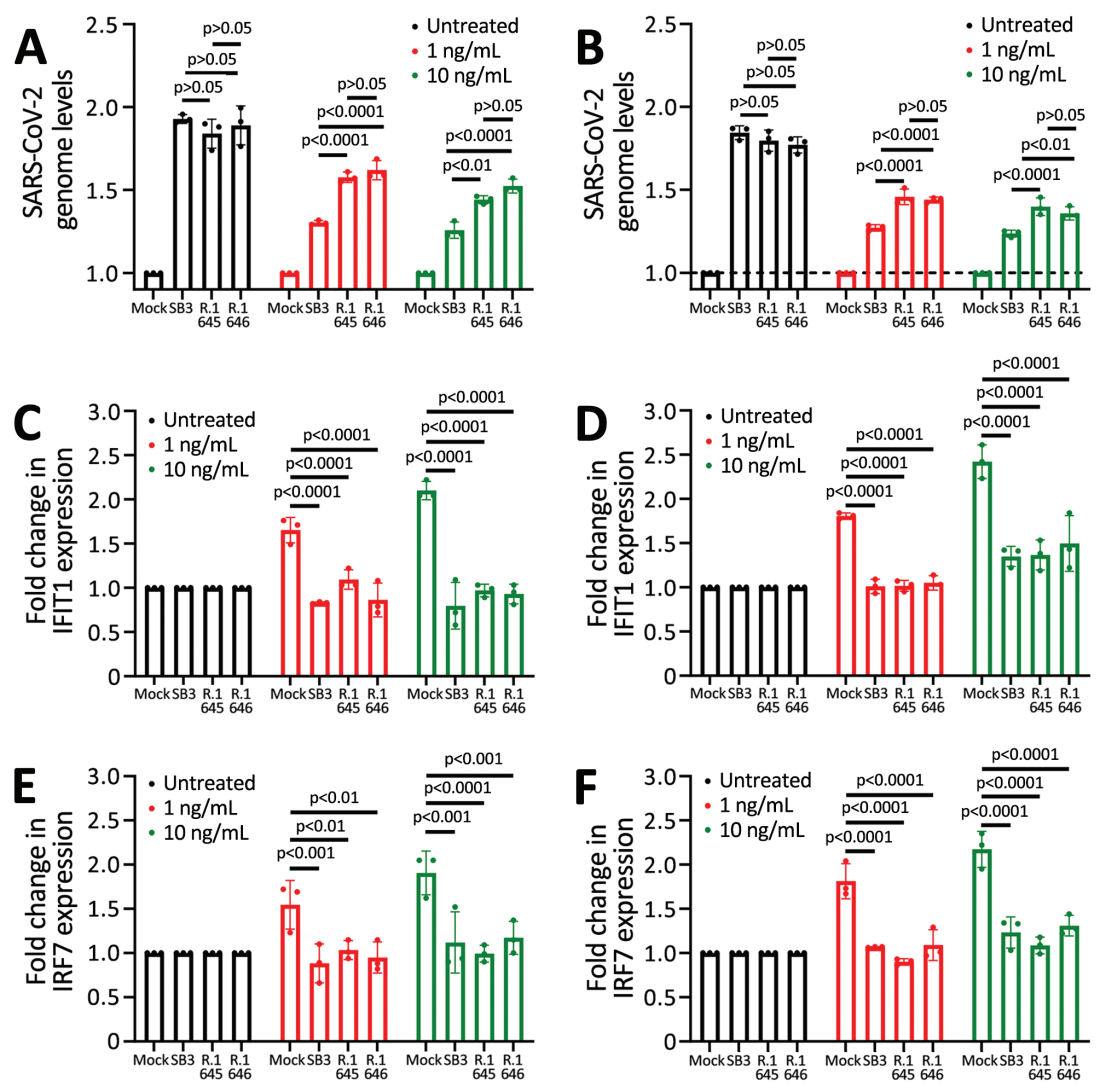
Resistance to type I interferons in SARS-CoV-2 R.1 lineage variants, Canada. A) Sensitivity of SB3, R.1 645, and R.1 646 to IFN-α. B) Sensitivity of SB3, R.1 645, and R.1 646 to IFN-β. C, D) Fold change in *IFIT1* transcript levels in response to IFN-α (C) or IFN-β (D) treatment. E, F) Fold change in *IRF7* transcript levels in response to IFN-α (E) or IFN-β treatment (F). ISG transcript levels were normalized to *GAPDH* transcript levels. For testing, Calu-3 cells were either mock-infected or infected with SARS-CoV-2 (50,000 PFU/well) for 1 hour followed by treatment with recombinant IFN (1 or 10 ng/mL). Total RNA was extracted after 72 hours and SARS-CoV-2 RNA levels were determined using quantitative reverse transcription PCR. 1/ΔCT values are represented after normalizing to mock-infected cells. Statistical significance was calculated using 2-way analysis of variance with Tukey multiple comparisons test. GAPDH, glyceraldehyde 3-phosphate dehydrogenase; IFIT1, interferon-induced protein with tetratricopeptide repeats; IFN, interferon; IRF7, interferon regulatory factor 7; ISG, IFN-stimulated gene.

Next, we investigated the sensitivity of R.1 isolates to type I IFNs. We infected Calu-3 cells with SB3 or one of the 2 R.1 isolates. One hour after absorption, we treated cells with IFN-α or IFN-β (1 ng/mL or 10 ng/mL) for 72 hours. We monitored SARS-CoV-2 RNA levels by using qRT-PCR after isolating total RNA from infected Calu-3 cells to determine the amounts of virus produced 72 hours after infection. As expected, we observed a drop in virus replication with recombinant IFN-α and IFN-β treatment (at both 1 ng/mL and 10 ng/mL concentration) (Figure 4, panels A, B). The R.1 isolates were significantly more resistant to both IFN-α and IFN-β than were SB3 (Figure 4, panels A, B). We observed no significant difference in SARS-CoV-2 RNA levels in the untreated controls. This finding indicates that R.1 isolate resistance to type I IFNs is not caused by differences in the level of incoming virus or inherent replication capacity.

We have previously shown that SARS-CoV-2 is a poor inducer of type I IFN and ISGs (*27*), and others have shown that SARS-CoV-2 can evade the type I IFN machinery (*28*,*29*). Because our R.1 isolates were significantly more resistant to type I IFNs, we examined differences in the expression of 2 ISGs: interferon-induced protein with tetratricopeptide repeats (IFIT1) and interferon regulatory factor 7 (IRF7). We compared the ability of the R.1 isolates to block IFIT1 and IRF7 production in response to IFN to that of SB3. We noted a dose-responsive increase in the transcript levels of *IFIT1* and *IRF7* for both IFN-α– and IFN-β–treated conditions in mock-infected cells (Figure 4, panels C–F). In the presence of SARS-CoV-2, we noted significant suppression of the IFN-α– and IFN-β–mediated activation of *IFIT1* and *IRF7* (Figure 4, panels C–F). However, we did not observe a significant difference in *IFIT1* or *IRF7* transcript levels between SB3 and the 2 R.1 isolates. We performed a viability assay to confirm that the difference in ISG signal is not caused by SARS-CoV-2–induced cell death (Appendix Figure 4, panels A, B). The absence of SARS-CoV-2–induced cytopathic effect corroborates our viability data (Appendix Figure 5, panels A, B) and is consistent with other studies (*30*,*31*). This observation implies that the resistance of the R.1 isolates to type-I IFNs is not inherently dependent on ISG modulation.

## Discussion

We demonstrate that R.1 isolates are sensitive to neutralizing antibodies induced after natural SARS-CoV-2 infection (Figure 3). These results are encouraging and add to our understanding of the sensitivity of VuMs to neutralizing antibodies. We further demonstrate that the R.1 lineage isolates, in contrast to the B.1.351 (Beta) VoC, retain neutralization sensitivity to antibodies generated early and later during the pandemic (Figure 3). Using a pseudovirus-based neutralization assay, a recent study demonstrated that mutations in R.1 lineage drive resistance to neutralizing antibodies compared with the wild type (*32*). Although a good correlation was observed between pseudovirus and live virus assay for antibody neutralization (*33*), a live virus assay more accurately represents spike protein density, epitope exposure, and replication kinetics of SARS-CoV-2. We observed a significant evolution in the breadth of neutralization with wave 3 antibodies, which resulted in a higher neutralization of R.1 isolates compared with the ancestral isolate (Figure 3, panels D–F). Of the 5 mutations in the spike region of our R.1 isolates, 3 are unique and not found in the B.1.351 (Beta) VoC. These include W_152_L and S_255_F substitutions in the N terminal domain of the S1 spike region and the G_769_V substitution in the S2 domain. A report that investigated key SARS-CoV-2 spike substitutions demonstrated that W_152_L alone does not confer neutralization resistance (*34*).

We established that the continuum of antibody-mediated neutralization is dependent on the virus inoculum (Figure 2, panels E, F) and that different levels of virus inoculum are neutralized differentially for SB3 and R.1 645. A significant drop in the ID_50_ titers was observed as we exponentially increased the number of SARS-CoV-2 particles. Those data imply that the threshold of neutralizing antibody titers necessary for protection is dependent on the exposure dose of virus particles. This finding indicates that a potent humoral response is critical for SARS-CoV-2 protection in vaccinated or naturally infected persons.

Our data show resistance of SARS-CoV-2 variants to type I IFNs, which in turn can influence viral evolution. An increased IFN resistance in SARS-CoV-2 was reported recently (*8*). Our R.1 isolates were significantly resistant to both IFNα and IFNβ treatment within lung epithelial cells (Figure 4, panels A, B). However, whether mutations accumulated in ORF1ab, ORF3, M, and N regions are causing the observed IFN resistance is unclear; this question will be vital in future studies. IFN resistance of R.1 isolates can potentially lead to higher viral loads, thereby accelerating virus shedding and transmission. Although the World Health Organization recategorized R.1 lineage isolates as formerly monitored variants in November 2021, data are limited on the transmission potential and disease severity of the formerly monitored variants. Our finding that R.1 lineage isolates are neutralization sensitive but concurrently IFN resistant indicates that IFN resistance will be a strong driving force in the generation of new variants. Overall, data from this study further advance our knowledge of how virus evolution can influence the trajectory and characteristics of a pandemic.

Appendix Additional information about sensitivity to neutralizing antibodies and resistance to type I interferons in SARS-CoV-2 R.1 lineage variants, Canada
