# Supplementary material for: Sensitivity to Neutralizing Antibodies and Resistance to Type I Interferons in SARS-CoV-2 R.1 Lineage Variants, Canada
Source: Emerg Infect Dis. 2023 Jul;29(7):1386–96. doi: 10.3201/eid2907.230198 (PMC10310370; doi:10.3201/eid2907.230198)
Supplement: Appendix — Additional information about sensitivity to neutralizing antibodies and resistance to type I interferons in SARS-CoV-2 R.1 lineage variants, Canada [file 23-0198-Techapp-s1.pdf]

Article DOI: https://doi.org/10.3201/eid2907.230198

EID cannot ensure accessibility for supplementary materials supplied by authors. Readers who have difficulty accessing supplementary content should contact the authors for assistance.

Sensitivity to Neutralizing Antibodies and Resistance to Type I Interferons in SARS-CoV-2 R.1 Lineage Variants, Canada

Appendix

Supplementary Methods

Cell Lines

Vero E6 cells (ATCC CRL-1586) were cultured in Dulbecco's Modified Eagle's media (DMEM) supplemented with 10% fetal bovine serum, 1x L-glutamine, and 1% penicillin-streptomycin. Calu-3 cells (ATCC HTB-55) were cultured in Minimum Essential Medium (Alpha MEM) supplemented with 10% fetal bovine serum, 1x L-glutamine, and 1% penicillin-streptomycin.

SARS-CoV-2 viruses

The SARS-CoV-2 ancestral isolate, SB3 was isolated and purified as described previously (*1*). The B.1.351 (β) VoC, isolate was obtained from BEI Resources (Manassas, VA, United States). The R.1 lineage variant was isolated in this study from patient nasopharyngeal swabs collected in universal transport media. Briefly, samples were diluted in equal proportion with DMEM containing 16 μg/ml TPCK-trypsin (Cat. No. 4370285, Sigma-Aldrich) and inoculated on VeroE6 cells seeded the previous day in a 12-well plate. After 1h adsorption, the inoculum was replaced with DMEM containing 2% FBS and 6 μg/mL TPCK-trypsin. The cells were observed daily for CPE and cell culture supernatants collected once positive for CPE. A 140 μl aliquot was used for viral RNA extraction using the Qiagen viral RNA extraction kit (Cat. No. 52904). The detection of SARS-CoV-2 RNA was performed using a qRT-PCR as previously described (*2*). $TCID_{50}$ assay was used to quantify the virus stock titers in Vero E6 cell (*1*).

Experiments with SARS-CoV-2 were performed in a biosafety containment level 3 facility and all procedures were approved by the institutional biosafety committee at McMaster University.

Human donors

Informed consent was obtained for the collection of convalescent serum from 39 patients with laboratory confirmed SARS-CoV-2 infection. The data on sex and age were collected from all the participants. This study was approved by the institutional review board for Sunnybrook Research Institute (REB#2218) and Sinai Health System (REB# 02–0118-U and 05–0016-C).

Sequencing and bioinformatic analyses

Sequencing of SARS-CoV-2 genomes from RNA extracts and subsequent bioinformatics analysis followed the steps detailed in Kotwa *et al* (*3*). Briefly, after cDNA synthesis and generation of ARTIC3 amplicons, DNA sequencing libraries were constructed for paired-end 300 bp sequencing (Illumina MiSeq) with ~2000x fold coverage. Short-read sequence processing and genome assembly was done using the SIGNAL (SARS-CoV-2 Illumina GeNome Assembly Line) pipeline (https://github.com/jaleezyy/covid-19-signal) to generate genome consensus, determine mutations relative to the ancestral Wuhan genome (MN908947.3), and to predict PANGOLIN lineage (*4*). Quality of consensus genome and overall sequencing was assessed using NCoV-Tools (https://github.com/jts/ncov-tools).

Phylogenetic tree

Dataset consisted of study sequences (R.1 645, R.1 646 and SB3), Los Alamos National Laboratories (LANL) full-length variant reference alignment from GISAID Web site (*5*), and randomly sampled α, β and gamma VoC sequences. Multiple sequence alignment was executed using MAFFT (*6*,*7*). A maximum likelihood (ML) tree was constructed with RaxML (*8*) by executing 1000 rapid bootstrap inferences and a thorough ML search using General Time Reversible model of nucleotide substitution. The tree was visualized using FigTree v1.4.2 (*9*).

Detection of SARS-CoV-2–specific binding antibodies

The IgG antibodies targeting the RBD were determined using the BioLegend LEGEND MAX SARS-CoV-2 Spike RBD Human IgG ELISA kit (Cat. No. 447707, BioLegend). The IgG antibodies targeting the spike S1 region were determined using the LEGEND MAX SARS-CoV-2 Spike S1 Human IgG ELISA kit (Cat. No.447807, BioLegend). The ELISA was performed according to the manufacturer’s instructions. Briefly, plates were washed four times with 300 μl

of 1x wash buffer. Next, human IgG standard were diluted 2-fold in assay buffer B. The standards ranged from 20 ng/ml to 0.313 ng/ml with 50 µl of standard per well. The serum samples were also diluted in assay buffer and 50 µl was added per well. The plates were sealed and incubated for 2 hours at room temperature with no shaking and washed four times with 300 µl of 1x wash buffer. Next, 100 µl of IgG detection antibody solution was added to each well. The plates were incubated at room temperature for 1 hour with no shaking and washed four times with 300 µl of 1x wash buffer. Next, 100 µl of Avidin-HRP solution was added to each well. The plates were incubated at room temperature for 1 hour with no shaking and washed five times with 300 µl of 1x wash buffer. Finally, 100 µl of substrate solution was added to each well and incubated for 10 minutes in the dark. The reaction was stopped by adding 100 µl of stop solution to each well. The absorbance was read at 450 nm within 10 minutes using the SpectraMax 190 microplate reader (Molecular Devices) at an optical density of 450 nm.

Molecular detection of SARS-CoV-2 RNA and $N_{501}Y$ mutation

Diagnostic nasopharyngeal or mid-turbinate swabs were collected from patients for SARS-CoV-2 testing and $N_{501}Y$ screening at Shared Hospital Laboratory (Toronto, Canada). RNA extraction and reverse transcription polymerase chain reactions (qRT-PCR) for SARS-CoV-2 detection were performed as previously described (*10*). In brief, RNA was extracted from 160 µl of sample using the MGISP-960 automated platform and the MGI Easy Magnetic Beads Virus DNA/RNA Extraction Kit (MGI Technologies, Shenzhen, China). qRT-PCR was performed using the CFX96 Touch Real-time PCR system (BioRad, Canada) with the envelope (E) gene, the 5′ untranslated region (5′ UTR), and the human RNase P internal control as targets. qRT-PCR for the $N_{501}Y$ screen was performed using the CFX96 Touch Real-time PCR (BioRad, Canada) and the Luna Universal Probe One-Step RT-qPCR Kit (New England Biolabs, Whitby, Ontario). Primer sequences used were: 501 Fwd – GAAGGTTTTAATTGTTACTTTC and 501 Rev – AAACAGTTGCTGGTGCATGT at 1.0 µM each. FAM-Y501 – CCAACCCACTTATGGTGTTG at 0.25 µM and HEX-N501 – CCAACCCACTAATGGTGTTG at 0.5 µM were the probe sequences used.

SARS-CoV-2 neutralization assay

Vero E6 cells were seeded at a density of $2.5x10^4$ cells per well in opaque 96-well flat-bottom plates in complete DMEM. Twenty-four hours later, patient serum was inactivated by incubating at 56°C for 30 minutes, then diluted 1:25 in low serum DMEM (supplemented with

2% FBS, 1x L-glutamine, and 1% penicillin-streptomycin). 2-fold serial dilution of the sample was performed in 96 well U-bottom plates. An equal volume of SARS-CoV-2 (15,000, 1,500 or 150 PFU/well) was then added to the diluted serum and the serum-virus mixture was incubated at 37°C for 1 hour. The Vero E6 culture media was then replaced with 100 µl of the serum-virus mixture and incubated at 37°C for 5 days. The plates were read by removing 50ul of culture supernatant and adding 50 µl of CellTiter-Glo 2.0 Reagent (Cat. No. G9243, Promega, Madison, WI, United States) to each well. The plates were then shaken at 282cpm at 3mm diameter for 2 minutes, incubated for 5 minutes at room temperature, then luminescence was read using a BioTek Synergy H1 microplate reader with a gain of 135 and integration time of 1 second.

Interferon treatment and quantitative PCR

Calu-3 cells were seeded at a density of $0.2x10^6$ cells/well in 12-well plates and infected with SARS-CoV-2 2 days post-seeding. The cells were either mock-infected or SARS-CoV-2 infected (50,000 PFU/well). Soon after the 1 h post-adsorption time, wells were washed twice with 1xPBS and treated with recombinant IFN-α (Cat. No. I4276, Sigma-Aldrich) or IFNβ (Cat. No. 300–02BC, PeproTech). RNA extraction was performed using RNeasy Mini Kit (Cat No. 74106, Qiagen) according to the manufacturer's protocol after 72 h post-infection. Five hundred nanograms of purified RNA were reverse transcribed using iScript gDNA Clear cDNA Synthesis Kit (Cat. No. 1725035, Bio-Rad). To quantify SARS-CoV-2 RNA qRT-PCR was performed using SsoFast EvaGreen supermix (Cat. No. 1725211, Bio-Rad) using the following primers: upE Fwd – ATTGTTGATGAGCCTGAAG and upE Rev – TTCGTACTCATCAGCTTG. To quantify ISGs, qRT-PCR reactions were performed with Taqman Universal PCR master mix using pre-designed Taqman gene expression assays (Thermo Fisher Scientific, Massachusetts, United States) for *GAPDH* (Cat. No. 4331182), *IFIT1* (Cat. No. 4331182) and *IRF7* (Cat. No. 4331182) according to manufacturer's protocol.

Viability assay

Calu-3 cells seeded in 12-well plates were either mock-infected or SARS-CoV-2 infected (1 hour exposure, 50,000 PFU/well), washed twice with sterile 1x PBS, and treated with recombinant IFN-α (Cat. No. I4276, Sigma-Aldrich) or IFNβ (Cat. No. 300–02BC, PeproTech). Two different concentrations of interferon were used for the assay (1ng/ml and 10 ng/ml). Cell viability was assessed by adding an equal volume of CellTiter-Glo 2.0 Reagent (Promega, Madison, WI, United States), mixed and lysates transferred to opaque 96-well flat-bottom plates

with six replicates per well. The plates were then shaken at 282cpm at 3mm diameter for 2 minutes and luminescence was read using a BioTek Synergy H1 microplate reader with a gain of 135 and integration time of 1 second.

Statistical analysis

All statistics were performed using Graph Pad Prism (Graph Pad Software, Inc., La Jolla, CA). An unpaired t test was used for Figure 2A, B. The correlations for Figure 2C, D and Appendix Figure 2, panels D–F were analyzed using Spearman r. One-way AVOVA with Tukey's multiple comparisons test was used to make comparisons in Figure 2E-G and Figure 3A-F. Two-way ANOVA with Tukey's multiple comparisons test was used for multiple comparisons in Figure 4. The ID_{50} values were calculated using a nonlinear regression model for Appendix Figure 1A-I and Appendix Figure 2A-C. The following p values were considered significant: $*p < 0.05$, $**p < 0.01$, and $***p < 0.001$, $****p < 0.0001$.

References

1. Banerjee A, Nasir JA, Budylowski P, Yip L, Aftanas P, Christie N, et al. Isolation, sequence, infectivity, and replication kinetics of severe acute respiratory syndrome coronavirus 2. Emerg Infect Dis. 2020;26:2054–63. PubMed https://doi.org/10.3201/eid2609.201495

2. Banerjee A, El-Sayes N, Budylowski P, Jacob RA, Richard D, Maan H, et al. Experimental and natural evidence of SARS-CoV-2-infection-induced activation of type I interferon responses. iScience. 2021;24:102477. PubMed https://doi.org/10.1016/j.isci.2021.102477

3. Kotwa JD, Jamal AJ, Mbareche H, Yip L, Aftanas P, Barati S, et al. Surface and air contamination with severe acute respiratory syndrome coronavirus 2 from hospitalized coronavirus disease 2019 patients in Toronto, Canada, March–May 2020. J Infect Dis. 2022;225:768–76. PubMed https://doi.org/10.1093/infdis/jiab578

4. Nasir JA, Kozak RA, Aftanas P, Raphenya AR, Smith KM, Maguire F, et al. A comparison of whole genome sequencing of SARS-CoV-2 using amplicon-based sequencing, random hexamers, and bait capture. Viruses. 2020;12:895. PubMed https://doi.org/10.3390/v12080895

5. Elbe S, Buckland-Merrett G. Data, disease and diplomacy: GISAID's innovative contribution to global health. Glob Chall. 2017;1:33–46. PubMed https://doi.org/10.1002/gch2.1018

6. Katoh K, Standley DM. MAFFT multiple sequence alignment software version 7: improvements in performance and usability. Mol Biol Evol. 2013;30:772–80. PubMed https://doi.org/10.1093/molbev/mst010

7. Katoh K, Misawa K, Kuma K, Miyata T. MAFFT: a novel method for rapid multiple sequence alignment based on fast Fourier transform. Nucleic Acids Res. 2002;30:3059–66. PubMed https://doi.org/10.1093/nar/gkf436

8. Stamatakis A. RAxML version 8: a tool for phylogenetic analysis and post-analysis of large phylogenies. Bioinformatics. 2014;30:1312–3. PubMed https://doi.org/10.1093/bioinformatics/btu033

9. Molecular evolution, phylogenetics and epidemiology [cited 2023 Feb 1]. http://tree.bio.ed.ac.uk

10. Kandel CE, Young M, Serbanescu MA, Powis JE, Bulir D, Callahan J, et al. Detection of severe acute respiratory coronavirus virus 2 (SARS-CoV-2) in outpatients: A multicenter comparison of self-collected saline gargle, oral swab, and combined oral-anterior nasal swab to a provider collected nasopharyngeal swab. Infect Control Hosp Epidemiol. 2021;42:1340–4. PubMed https://doi.org/10.1017/ice.2021.2

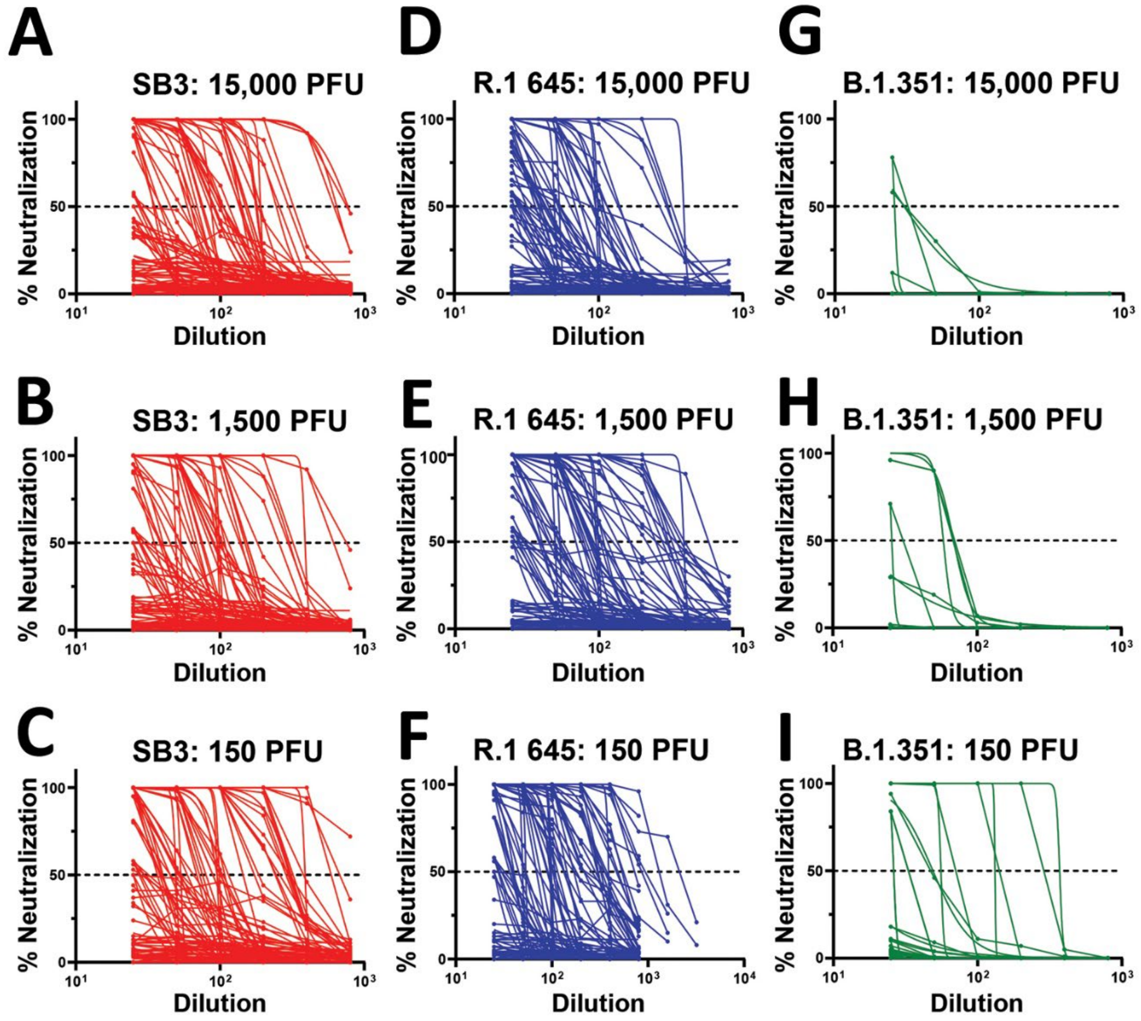

Appendix Figure 1. Regression line depicting the neutralization activity of the 39 convalescent serum samples tested on the three SARS-CoV-2 isolates. A PFU/well of 15,000, 1,500 and 150 were used for each of the SARS-CoV-2 isolates. (A-C) Neutralization profile of SB3. (D-F) Neutralization profile of R.1 645. (G-I) Neutralization profile of B.1.351 (Beta) VoC.

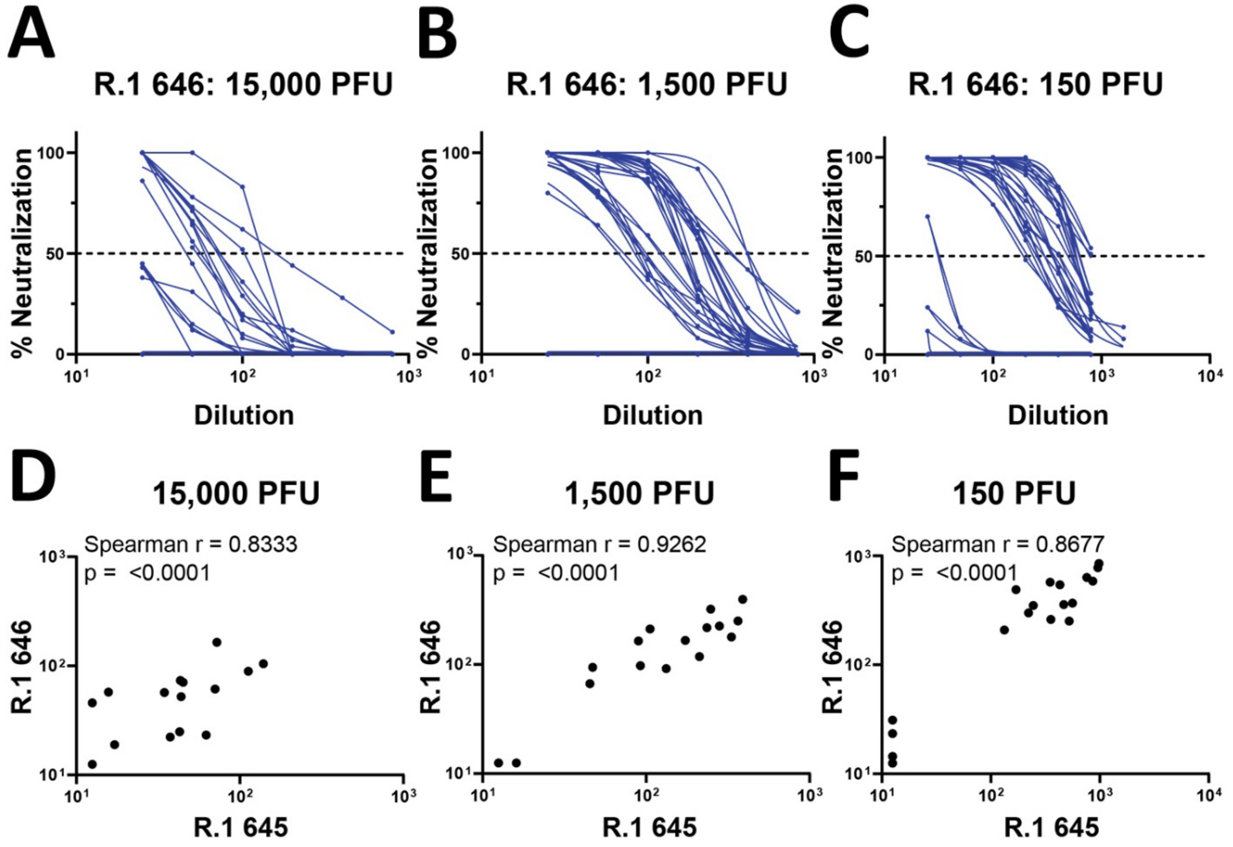

Appendix Figure 2. (A-C) Regression line depicting the neutralization activity of a subset of the convalescent serum samples (n = 19) to R.1 646. A PFU/well of 15,000, 1,500 and 150 were used. (D-F) ID_{50} titer correlation between R.1 645 and R.1 646 for 15,000, 1,500 and 150 PFU/well.

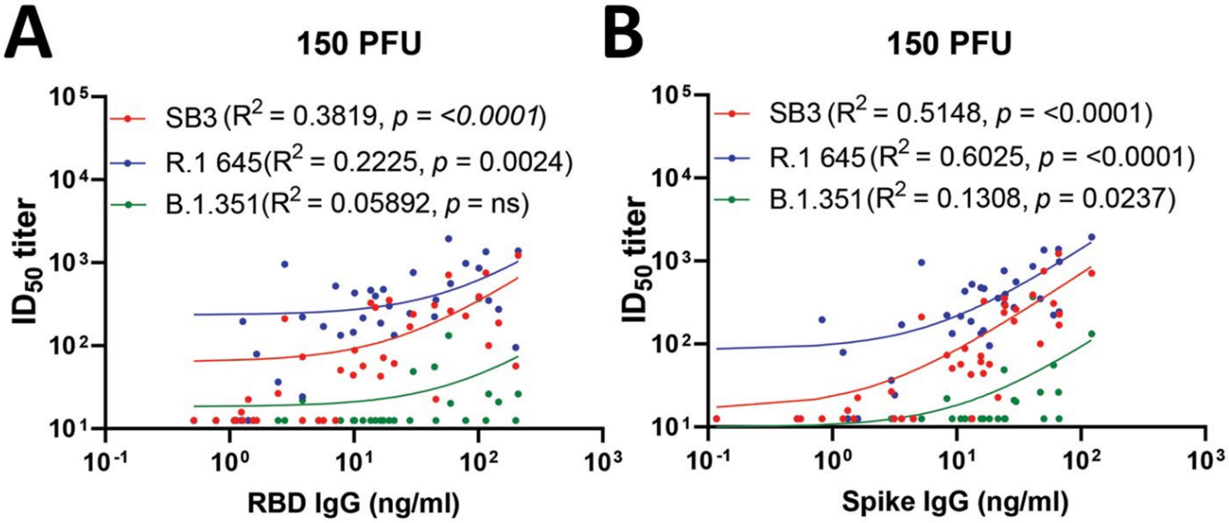

Appendix Figure 3. (A) Predicting neutralization of SB3, R,1 645 and B.1.351 (Beta) VoC (150 PFU/well) using RBD binding IgG antibodies. (B) Predicting neutralization of SB3, R,1 645 and B.1.351 (Beta) VoC (150 PFU/well) using spike (S1) binding IgG antibodies. The R-squared values were calculated using a simple linear regression model. (ns-not significant)

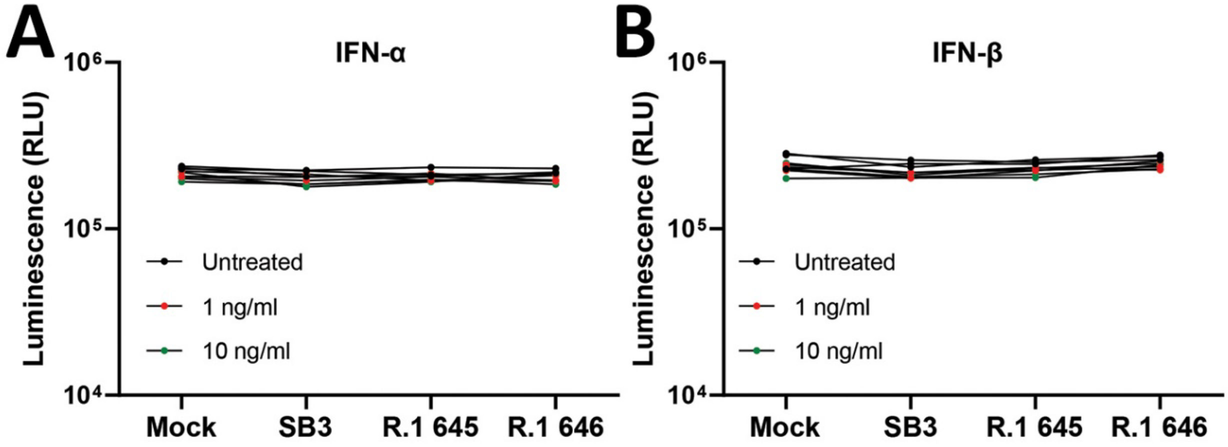

Appendix Figure 4. (A, B) Calu-3 cells were either mock-infected or infected with SARS-CoV-2 (50,000 PFU/well) for 1 h followed by treatment with recombinant IFNα (1 or 10 ng/mL) or IFNβ (1 or 10 ng/mL). Seventy-two hours later, viability was assessed using CellTiter-Glo 2.0 reagent and luminescence measured using a BioTek Synergy H1 microplate reader.

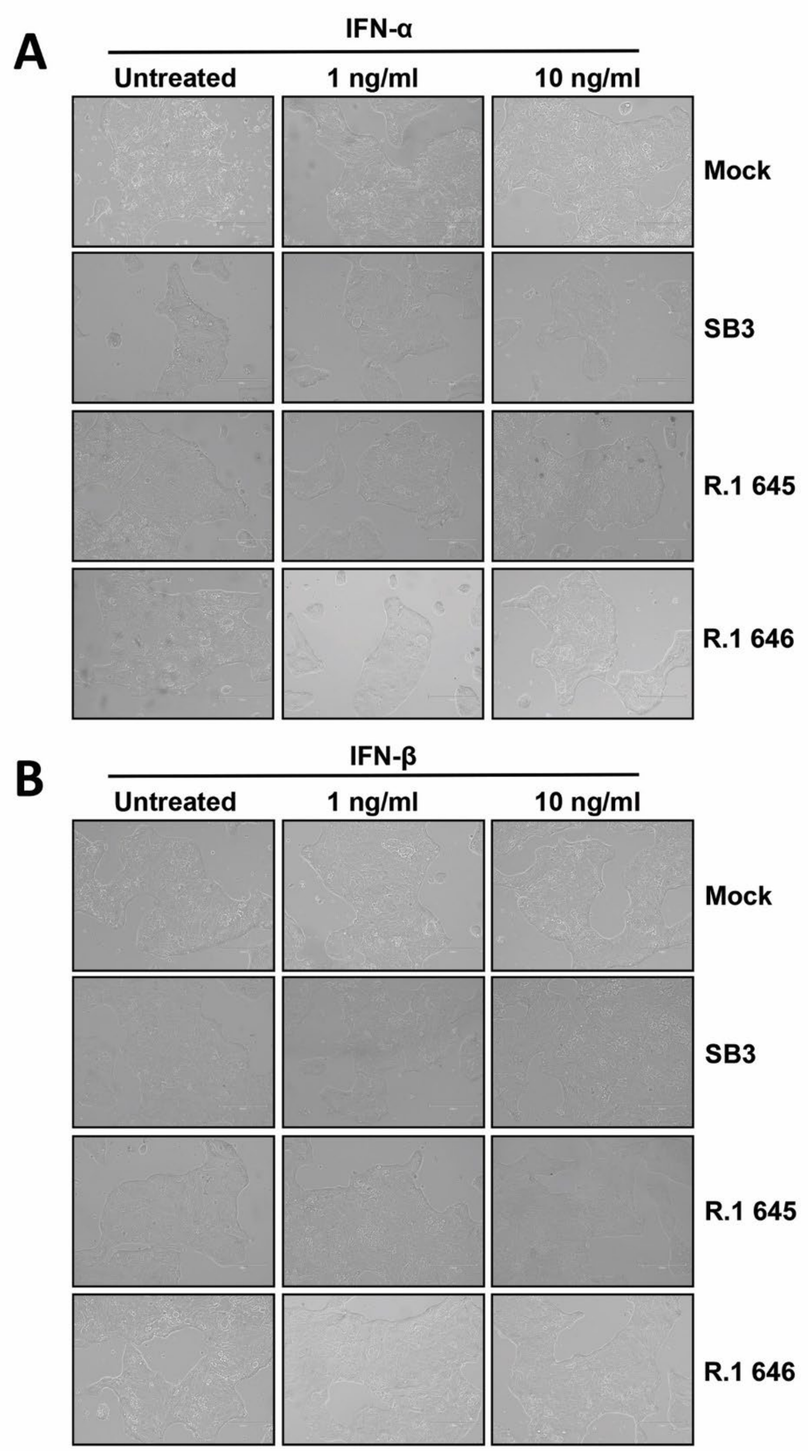

Appendix Figure 5. (A, B) Calu-3 cells were either mock-infected or infected with SARS-CoV-2 (50,000 PFU/well) for 1 h followed by treatment with recombinant IFNα (1 or 10 ng/mL) or IFNβ (1 or 10 ng/mL). Seventy-two hours later, cells were imaged using a EVOS M5000 microscope. Images shown are representative cells from three replicates for each condition. The scale bar is 300 μm.
